# Which resources should be used to identify RCT/CCTs for systematic reviews: a systematic review

**DOI:** 10.1186/1471-2288-5-24

**Published:** 2005-08-10

**Authors:** Ellen T Crumley, Natasha Wiebe, Kristie Cramer, Terry P Klassen, Lisa Hartling

**Affiliations:** 1 HealthInfo & Searching Practice Inc., Edmonton, Canada; 2 Department of Medicine, Division of Nephrology, University of Alberta, 4058 Research Transition Facility, Edmonton, Alberta T6G 2E1, Canada; 3 Department of Pediatrics, Complementary and Alternative Research and Education (CARE) Program, University of Alberta, 4047 Research Transition Facility, Edmonton, Alberta T6G 2E1, Canada; 4 Department of Pediatrics, Alberta Research Centre for Child Health Evidence (ARCHE), University of Alberta, 4^th ^Floor Aberhart Centre One, 11402 University Avenue, Edmonton, Alberta T6G 2J3, Canada

## Abstract

**Background:**

Systematic reviewers seek to comprehensively search for relevant studies and summarize these to present the most valid estimate of intervention effectiveness. The more resources searched, the higher the yield, and thus time and costs required to conduct a systematic review. While there is an abundance of evidence to suggest how extensive a search for randomized controlled trials (RCTs) should be, it is neither conclusive nor consistent. This systematic review was conducted in order to assess the value of different resources to identify trials for inclusion in systematic reviews.

**Methods:**

Seven electronic databases, four journals and Cochrane Colloquia were searched. Key authors were contacted and references of relevant articles screened. Included studies compared two or more sources to find RCTs or controlled clinical trials (CCTs). A checklist was developed and applied to assess quality of reporting. Data were extracted by one reviewer and checked by a second. Medians and ranges for precision and recall were calculated; results were grouped by comparison. Meta-analysis was not performed due to large heterogeneity. Subgroup analyses were conducted for: search strategy (*Cochrane*, *Simple*, *Complex*, *Index*), expertise of the searcher (Cochrane, librarian, non-librarian), and study design (RCT and CCT).

**Results:**

Sixty-four studies representing 13 electronic databases met inclusion criteria. The most common comparisons were MEDLINE vs. handsearching (n = 23), MEDLINE vs. MEDLINE+handsearching (n = 13), and MEDLINE vs. reference standard (n = 13). Quality was low, particularly for the reporting of study selection methodology. Overall, recall and precision varied substantially by comparison and ranged from 0 to 100% and 0 to 99%, respectively. The trial registries performed the best with median recall of 89% (range 84, 95) and median precision of 96.5% (96, 97), although these results are based on a small number of studies. Inadequate or inappropriate indexing was the reason most cited for missing studies. *Complex *and *Cochrane *search strategies (SS) performed better than *Simple *SS.

**Conclusion:**

Multiple-source comprehensive searches are necessary to identify all RCTs for a systematic review, although indexing needs to be improved. Although trial registries demonstrated the highest recall and precision, the *Cochrane *SS or a *Complex *SS in consultation with a librarian are recommended. Continued efforts to develop CENTRAL should be supported.

## Background

The aim of systematic reviews is to present the most valid estimate of the effectiveness of the intervention in question. To do so, the identification of relevant studies must be comprehensive and unbiased. Systematic reviews usually include a comprehensive summary of data from both randomized (RCT) and controlled trials (CCT), although other study designs are sometimes incorporated. There is an ongoing debate about the number and type of resources that should be used to identify trials for systematic reviews [1-3]. These resources include electronic databases, the Internet, handsearching, checking relevant article references, and personal communication with experts in the field. Reviewers are encouraged to search numerous resources in order to identify as many relevant studies as possible without systematically introducing bias [4]. However, searching more resources typically results in a higher yield; thus, more time and resources are required to conduct the review [5]. Consequently, determining the relative value of different sources of trials is critical to enhance the efficiency of systematic reviews, while maintaining their validity.

The Cochrane Collaboration Reviewers' Handbook notes that MEDLINE, EMBASE, and CENTRAL are the three electronic bibliographic databases generally considered as the richest sources of trials [6]. The Cochrane Collaboration maintains that handsearching is vital to the credibility and success of systematic reviews [7]. Hopewell et al. conducted a systematic review of studies that compared handsearching to searching an electronic database for RCTs [8]. In the 34 included studies, they found that complex searches of electronic databases recalled 65% of relevant RCTs; the other 35% were retrieved in other ways including handsearching. They concluded that "handsearching still has a valuable role to play in identifying reports of randomized controlled trials for inclusion in systematic reviews of health care interventions, particularly in identifying trials reported as abstracts, letters and those published in languages other than English, together with all the reports published in journals not indexed in electronic databases" [8].

Our research question was: Does resource-specific searching retrieve RCT/CCTs for systematic reviews with the same recall and precision as those searches which combine two or more distinct resources? Our primary goal was to identify and quantitatively review studies comparing two or more different resources (e.g., databases, Internet, handsearching) used to identify RCTs and CCTs for systematic reviews. Specifically, we were interested in determining the value (in terms of identifying unique citations) of searching key resources (e.g., EMBASE, CENTRAL, PsycINFO, handsearching) in addition to the key resource MEDLINE.

## Methods

### Search strategy

Seven electronic databases (MEDLINE, EMBASE, CINAHL, ERIC, PsycINFO, Web of Science, Cochrane Library) were searched from their inception to April 2004. The MEDLINE search strategy was tailored as necessary for each database (Appendix 1). Four journals were handsearched from 1990 to 2004: Health Information & Libraries Journal (Health Libraries Review), Hypothesis, Journal of the Medical Library Association (Bulletin of the Medical Library Association), Medical Reference Services Quarterly. All abstracts presented at Cochrane Colloquia (1993–2003) were handsearched. In addition, key authors were contacted via email and references of relevant articles were screened. The searches were not limited by language or date of publication. Searches are available upon request from the corresponding author.

### Study selection

Two reviewers independently screened the yield from the searches to identify potentially relevant studies. The full text of these studies was obtained and two reviewers independently applied inclusion/exclusion criteria using a standard form. Any differences were resolved through discussion. Studies were included if they compared two or more sources to find RCTs or CCTs (e.g., one or more resources compared against a "gold standard"; handsearch versus MEDLINE; and, EMBASE versus MEDLINE). Inclusion was not limited by the topic/content area in the individual studies.

A study was excluded if: it only compared different search strategies within the same database; it only included non-randomized trials; or, if the resource is not currently accessible. If authors searched for all study designs including trials, it was included only if data were reported separately for RCTs or CCTs.

RCTs were defined as an experiment in which eligible patients are assigned to two or more study groups using an appropriate method of randomization [9]. CCTs were defined similarly except that the method of allocation is not necessarily random. When authors did not provide definitions, we accepted their classification/indication of study design.

### Assessment of quality

We developed a checklist to assess the quality of reporting of the included studies. The quality items were chosen based on threats to the validity of comparative studies that have been empirically supported in the literature [10]. The items assessed reporting in four key areas:

• Was there an adequate description of what the search was attempting to identify (e.g., type of studies, content area, standard inclusion/exclusion criteria);

• Was there an adequate description of the methods used to search (e.g., resource(s), words/subject headings used, time period covered, date of search);

• Was there an adequate description of the reference standard (e.g., how many references) and how it was derived (e.g., sources searched and methods used); and,

• Was bias avoided in the selection of relevant studies (e.g., was there an independent assessment of studies for inclusion by more than one researcher).

Two reviewers independently applied the checklist to the included studies. Discrepancies were resolved through discussion.

### Data extraction

Data were extracted independently by one investigator and checked by a second independent investigator. A standard form was used to extract the following information: language of publication, country where study was conducted, year of publication, study design and objective(s), resources being compared, topic being searched, years the search covered, search strategies used, results (yield, recall, precision, reasons studies were missed), and author's conclusions. When data were not available, authors were contacted and asked to supply the missing information.

### Data analysis

Data were analyzed using Splus 6.2 (Insightful Corporation 2003). Recall and precision were expressed as percentages with 95% confidence intervals which were calculated using exact methods [11]. Recall is the percentage of relevant studies that were identified by the search. Precision is the percentage of studies identified by the search that were relevant. Results were grouped by comparison (e.g., MEDLINE versus handsearching, MEDLINE vs. other reference standard). Meta-analysis was not performed due to large heterogeneity. Comparisons, however, were summarized using medians and ranges. With regards to the independence of the results, we conducted a sensitivity analysis around the inclusion of duplicate topics. Duplicate topics for exclusion were randomly selected and the median and range summaries were re-calculated.

Possible sources of heterogeneity were explored with numerical summaries both by within-study and between-study analyses. Within-study analyses are direct analyses and they occur when two searches are conducted with the same known conditions (i.e., strategy, expertise of the searcher, topic of the search, type of design) and some unknown conditions except for the condition or variable of interest. These are also called direct analyses. Between-study analyses are indirect analyses and are of a lower grade [12] in that there is a stronger potential for known variables (i.e., topic, strategy, author of search, topic of search, type of design) to confound results. The variables of interest we explored were: search strategy (*Cochrane*, *Simple*, *Complex*, *Index*), expertise of the searcher (Cochrane, librarian, non-librarian), and study design (RCT and CCT).

Searches were divided into the following four categories (modified from Hopewell et al. [8]): *Complex *(using a combination of types of search terms); *Cochrane *(the Cochrane Highly Sensitive Search Strategy (HSSS)); *Simple *(using five or fewer search terms which may include a combination of MeSH, Publication Type, keywords); and, *Index *(using one or two search terms (usually author's name or article title) to check/verify if the study is in a database).

## Results

### Description of included studies

**Figure 1:**
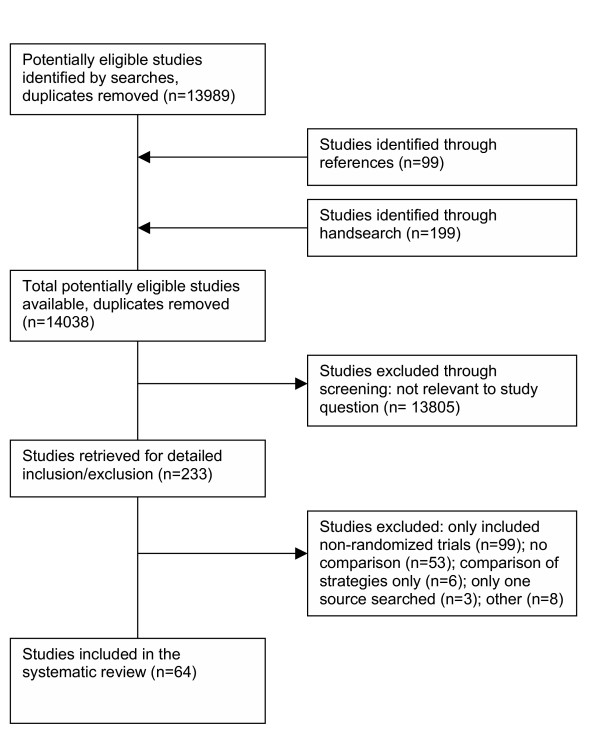
Quorum flow diagram.

Sixty-four studies met the criteria for inclusion in this analysis (Figure 1; see Additional file 1). Of these, 49 were published as full manuscripts, 12 were abstracts, 2 were letters, and 1 was a conference presentation. All studies were published between 1985 and 2003 with 94% being published after 1988, the same year MEDLINE became freely available through the PubMed interface . Approximately half the studies (n = 30) were conducted in the United Kingdom. Three studies were non-English (German, Dutch and Spanish). Thirty studies received funding, some from more than one source. Financial support was received from: 16 government programs, 2 pharmaceutical companies and 35 other sources (e.g., Universities, health trusts, foundations/associations, the National Library of Medicine and individual journals).

The included studies searched a variety of topics which fell into four major categories: journal (e.g., Lancet, BMJ), disease/condition/state (e.g., hepatitis, rheumatoid arthritis), specialty/sub-specialty (e.g., rehabilitation, pediatrics) and methodology (e.g., search strategies) [see Additional file 1]. Generally, the objectives of the studies were: to compare different searches (e.g., handsearch vs. database); to determine recall/precision of a search; to handsearch a journal and check to see if trials were in a database; or to develop a trial register.

The reference standards varied (e.g., handsearching, handsearching plus MEDLINE, MEDLINE plus EMBASE plus other databases). The specific study design for which authors were searching varied by study: RCTs only (n = 27); RCTs and CCTs (n = 28); and RCTs, CCTs, and other designs (n = 9).

There were four major comparisons: MEDLINE vs. handsearch (n = 22), MEDLINE vs. MEDLINE + handsearch (n = 12), MEDLINE vs. other reference standard (n = 18), and EMBASE vs. reference standard (n = 13). There were 13 other comparisons with only one to two studies each (Table 1).

**Table 1:** Results

**Comparison**	**Recall**			Precision		
	**Number of Studies**^†^	**Number of Comparisons**	**Median (Range)**	**Number of Studies**^†^	**Number of Comparisons**	**Median (Range)**

MEDLINE vs. Handsearching	23	41 22*	53 (7,97) 58 (7,97)	12	23 11	35 (0.03,99) 31 (0.03,78)
MEDLINE vs. MEDLINE + Handsearching	13	16 12*	70 (18,90) 75 (18,90)	6	8 5	49 (13,83) 40 (13,83)
MEDLINE vs. Reference Standard	13	24 18*	59 (17,98) 58 (17,98)	8	17 11	27 (1,91) 30 (1,91)
EMBASE vs. Handsearching	2	2	(42,88)	2	2	(9,17)
EMBASE vs. Reference Standard	9	14 13*	72 (13,100) 68 (13,100)	5	8 7	28 (0,48) 26 (0,48)
PsycINFO vs. Handsearching	2	2	(68,70)	2	2	(8,9)
PsycINFO vs. Reference Standard	2	4 2*	50 (0,65) (38,61)	2	3 2	39 (36,47) (36,47)
Trial Registries vs. Reference Standard	2	4	89 (84,95)	1	2	(96,97)
CINAHL vs. Reference Standard	2	2	(0,1)	1	1	0
Biosis vs. Reference Standard	1	1	47	1	1	1
CancerLIT vs. Handsearching	1	1	92	0	--	--
Cabnar vs. Reference Standard	1	1	47	1	1	2
CENTRAL vs. Reference Standard	1	1	78	0	--	--
Chirolars vs. Reference Standard	1	1	49	0	--	--
HealthStar vs. Reference Standard	1	1	53	1	1	1
Internet vs. Reference Standard	1	1	24	1	1	17
SciCitIndex vs. Reference Standard	1	1	61	0	--	--

* excludes duplicate topics

**† **excludes *Index *searches

### Methodological quality of included studies

All studies indicated the type of study design for which they were searching and all but one specified the topic area. Most (70%) stated their inclusion/exclusion criteria. Eighty percent of the studies described (or indicated they were available) reproducible search strategies/methods. Half of the studies stated who developed the search strategies and of these only 2 did not provide reproducible information about their search strategy. Eighty-five percent of articles fully described how the reference standard was compiled. Twenty-five percent reported that at least 2 people independently screened searches and in 35% at least 2 people also independently applied eligibility criteria.

### Quantitative results

Table 1 summarizes the results of the comparisons (e.g., MEDLINE versus handsearching, MEDLINE vs. other reference standard). Thirteen databases (including the Internet) were included in the numerical results. The results from 7 studies could not be used in the data analysis for the following reasons: did not use same journals for handsearch and MEDLINE [13]; insufficient data available (usually because it was an abstract) [14-18]; reporting flaw(s) which could not be clarified by author(s) [19].

### MEDLINE

Forty-nine studies had usable data for the MEDLINE comparisons. Three comparisons were analyzed: MEDLINE versus handsearching (41 comparisons, 23 studies), MEDLINE versus MEDLINE plus handsearching (16, 13), and MEDLINE versus other reference standards (24, 13). Estimates of both recall and precision for all three comparisons varied substantially, ranging from 7 to 98% and 0.03 to 99%, respectively. The estimates for MEDLINE versus handsearching and MEDLINE versus a reference standard were comparable: median of 53 versus 59% for recall and 35 versus 27% for precision. Median recall and precision for MEDLINE versus MEDLINE plus handsearching were somewhat larger (70 and 49%, respectively).

### EMBASE

Eleven studies had usable data for the EMBASE comparisons. Two comparisons were analyzed: EMBASE versus handsearching (2 comparisons, 2 studies) and EMBASE versus a reference standard (14, 9). Individual study estimates ranged from 13 to 100% for recall and 0 to 48% for precision. Summarizing all studies, medians were 65 and 72% for recall and, 13 and 28% for precision.

### PsycINFO

Four studies contained data for the PsycINFO comparisons. Two comparisons were analyzed: PsycINFO versus handsearching (2 comparisons, 2 studies), and PsycINFO versus a reference standard (4, 2). Recall ranged from 0 to 70%. Precision ranged from 8 to 47%. Medians were 69 and 50% for recall and 9 and 39% for precision.

### Other databases

Two studies investigated CINAHL and trial registers. Only one study had usable data from other databases (i.e., BIOSIS, CancerLit, CABNAR, CENTRAL, Chirolars, HealthSTAR, the Internet, SciCitIndex). The results from the trial registries versus a reference standard were consistent and high: 89% for median recall and 97% for median precision. The remaining comparisons ranged from 0 to 92% for median recall and 0 to 17% for median precision. Regardless of topic, there were too few included studies in these comparisons for this data to be representative.

### Subgroup analyses

Table 2 shows the results for the direct subgroup analyses. Seven studies were included in the search strategy analysis. There were six comparisons of *Simple *versus *Complex *search strategies. All but one study [20] showed greater recall for the *Complex *search strategies. The trade-off which so often occurs between recall and precision did not occur: three out of the four *Complex *search strategies had larger (better) precision (not including Fergusson [20]).

**Table 2:** Direct Subgroup Analyses

Study	Recall (95% CI)		Precision (95% CI)	
***Simple versus Complex***				

	Simple	Complex	Simple	Complex

Adams[26] 1994	18 (15,21)	52 (48,56)	40 (35,46)	59 (55,63)
Bender[27] 1997	53 (47,58)	65 (59,70)	--	78 (73,83)
Dickerson[28] 1985a	18 (10,26)	29 (20,39)	65 (45,86)	72 (56,87)
Dickerson[28] 1985b	32 (15,50)	56 (38,74)	38 (19,57)	53 (35,70)
Fergusson[20] 2000	89 (81,97)	88 (80,96)	1 (1,1)	3 (2,4)
Marson[29] 1996	64 (55,73)	86 (80,93)	72 (63,81)	35 (29,40)

***Simple versus Cochrane***				

	Simple	Cochrane	Simple	Cochrane

Brand[30] 1998a	59	93	--	--
Brand[30] 1998b	88	97	--	--
Fergusson[20] 2000	89 (81,97)	89 (81,97)	1 (1,1)	7 (6,9)
McDonald[31] unpub a	62 (55,68)	76 (70,82)	--	--
McDonald[31] unpub b	52 (43,61)	91 (86,96)	--	--

***Complex versus Cochrane***				

	Complex	Cochrane	Complex	Cochrane

Fergusson[20] 2000	88 (80,96)	89 (81,97)	3 (2,4)	7 (6,9)

***Author versus Librarian***				

	Author	Librarian	Author	Librarian

Kirpalani[21] 1989	34 (20,48)	53 (38,67)	--	--

There were five direct comparisons of a *Simple *search strategy versus the *Cochrane *HSSS. Again, all but one of the comparisons [20] had larger sensitivities for the *Cochrane *search strategy. None of these four comparisons reported precision. Fergusson [20] found negligible differences for both recall and precision.

Three MEDLINE comparisons were considered for the indirect comparisons (Tables 3 and 4) since they were the only ones sizable in number. Our indirect results differed from the direct results. No systematic differences between the *Simple *and *Complex *search strategies in median recall were found: 49 versus 51% for MEDLINE versus handsearching, 48 versus 67% for MEDLINE versus MEDLINE plus handsearching, and 58 versus 40% for MEDLINE versus a reference standard. The precision results were similar: 76 versus 38%, 62 versus 51%, and 23 versus 35%, respectively. And although the median precision estimates for the *Cochrane *search were much smaller (9, 48, and 7%), the median recall estimates (67, 81, and 78%) were systematically greater when compared to the *Simple *and *Complex *search strategies.

**Table 3:** Indirect Subgroup Analyses: Recall

**Subgroups**		**MEDLINE vs. Handsearching**		**MEDLINE vs. MEDLINE + Handsearching**		**MEDLINE vs. Reference Standard**	
		N	Median (Range)	N	Median (Range)	N	Median (Range)

**Search Strategy**	Index	6	93 (41,100)	0		1	66
	Cochrane	12	67 (26,97)	7	81 (28,90)	6	78 (53,98)
	Complex	15	51 (9,86)	6	67 (52,88)	10	40 (17,97)
	Simple	14	49 (7,88)	3	48 (18,72)	8	58 (18,89)
**Author**	Index	6	93 (41,100)	0	--	1	66
	Cochrane	12	67 (26,97)	7	81 (28,90)	6	78 (53,98)
	Librarian	5	49 (20,62)	0	--	3	56 (29,89)
	Non-librarian	24	52 (7,88)	9	66 (18,88)	15	48 (17,97)
**Design**	RCT	19	54 (9,97)	1	28	10	79 (25,97)
	CCT	28	62 (7,100)	15	72 (18,90)	15	56 (17,98)

**Table 4:** Indirect Subgroup Analyses: Precision

**Subgroups**		**MEDLINE vs. Handsearching**		**MEDLINE vs. MEDLINE + Handsearching**		**MEDLINE vs. Reference Standard**	
		N	Median (Range)	N	Median (Range)	N	Median (Range)

**Search Strategy**	Cochrane	7	9 (0.03,76)	3	48 (13,49)	3	7 (1,7)
	Complex	12	38 (22,78)	3	51 (13,59)	9	35 (3,91)
	Simple	4	76 (68,99)	2	(40,83)	5	23 (1,65)
**Author**	Cochrane	7	9 (0.03,76)	3	48 (13,49)	3	7 (1,7)
	Librarian	1	34	0	--	2	(53,72)
	Non-librarian	15	50 (22,99)	5	51 (13,83)	12	29 (1,91)
**Design**	RCT	12	43 (22,79)	0	--	7	7 (1,91)
	CCT	11	28 (0.03,99)	8	49 (13,83)	10	33 (7,72)

Only one study [21] directly compared search strategies from two different authors (i.e., a librarian versus a non-librarian). In this one example, the librarian's *Complex *search had a recall of 53% and the non-librarian's *Complex *search had a recall of 34. For the indirect subgroup comparisons, the librarians did not systematically outperform the non-librarians on either median recall or median precision; however the *Cochrane *HSSS (as author) did outperform the librarians and non-librarians on median recall (67, 81 and 78%).

No studies directly compared searching for RCTs versus CCTs. Based on indirect comparisons, the three MEDLINE comparisons showed no systematic difference in median recall and precision between design types.

A sensitivity analysis excluding duplicate topics was performed due to the concern for non-independence between studies. Studies or comparisons searching on the same topic may include some of the same relevant studies. We picked one comparison randomly from each topic area and eliminated it from the main quantitative results. The results are shown in Table 1. We found that recall had similar ranges and medians signifying that non-independence was not distorting our results.

### Reasons for missed trials

Table 5 lists the most common reasons articles were missed in both the electronic and handsearches. Forty-two studies reported reasons for missing studies from the search or handsearch. For electronic databases, the reason cited most often (67%) for missed studies was inadequate or inappropriate indexing. Other major reasons why studies were not found in databases included: they were published as abstracts, books, book reviews, brief reports, letters, proceedings or supplements, etc. (i.e., grey literature) (21%); keywords or methodology were not reported by author (21%); insufficient or restricted search strategy (14%); article(s) were omitted or missing from a resource (14%).

**Table 5:** Reasons why studies were missed by electronic search and handsearch

**Category**	**Reason**	**Studies citing reason (n = 42)**
*Electronic Resources*		
Indexing		
	Inadequate indexing in general	16[3,27,29,32–44]
	Journal not indexed in resource	8[3,13,20,27,35,40,45,46]
	Not indexed with proper methodologic terms	9[21,26,28,29,32,36,42,47,48]
	Not indexed as RCT/CCT	7[35,37,49–53]
	Not indexed for any of the methodologic terms used by searchers	2[21,54]
	Database does not contain relevant index terms	2[17,50]
	Not indexed with any of the subheadings used in searches	1[21]
Search Strategy		
	Insufficient or restricted search strategy	6[27,32,35,55–57]
Reporting		
	Keywords or methodology not reported by author	9[13,19,29,34,41,48,53,58,59]
Database		
	Issue(s) omitted from resource	4[3,26,56,60]
	Time lag in updating resource	2[60,61]
	Article(s) omitted or missing from resource	6[3,29,36,50,51,56]
Journal		
	Published before database was created or beyond coverage years of database	2[56,60]
Other		
	Unknown reason	6[26,38,38,54,60,62]
	Published as abstracts, books, book reviews, brief reports, letters, proceedings, supplements, etc.	9[20,26,38,38,49,51,56,63]
		• Wrong abstract assigned to reference[36] • Journals do not encourage authors to explicitly report methodology[37] • Length and complexity of search is limited using search engines[64] • Ongoing or recently finished studies[64] • Found through references[20] • Missed most Japanese reports[65] • Some personal communication did not get responses; organizations and people must be willing to supply trial information[18] • Not all abstracts are published in full[50] • Authors misspelled gingko[39] • Many RCTs are not identifiable in database[66]
*Handsearch*		
	Handsearchers not trained properly	2[57,67]
	Methodologic terms "hidden" in article	1[26]
	Searcher fatigue, boredom	1[26]
	Journal not handsearched	2[35,42]
	Article not an RCT or CCT	1[54]
Other		
	Different topic than what handsearchers were looking for	1[42]

In this study, seven studies performed *Index *searches. Six comparisons using the *Index *searches were found for MEDLINE versus handsearching. The median recall was 93% and the range was 41 to 100%. On average, 7% of studies were not indexed adequately. One study compared MEDLINE to a reference standard, their *Index *search produced 66% of the included studies. Two further *Index *searches were performed: EMBASE versus handsearching and PsycINFO versus handsearching; their recalls were 52 and 97%, respectively.

For handsearching, few authors provided information for why trials were missed. Handsearches had high precision and some studies did not miss any references through their handsearches. In the 3 studies where handsearchers missed studies, authors reported the reasons for missing studies were the handsearchers were not trained properly or they had fatigue/boredom. In two studies where trials were missed, authors reported that the journal was not handsearched, yet a database was used to search for this same journal. One of the missed articles was misclassified by a handsearcher as an RCT/CCT and one had a different topic than what handsearchers were meant to identify. Results from the MEDLINE versus MEDLINE plus handsearching comparisons quantify the percentage of trials missed by handsearching. In 13 studies, the median percentage found in MEDLINE but not by handsearching was 6% (range 1 to 15%).

## Discussion

For certain topics trial registries may be sufficient (e.g., perinatology, Japanese), however, the median recall estimates (Table 1) were not large enough to support single-source searches. These data highlight the importance of searching multiple sources when conducting a systematic review. Initiatives to compile references from different sources, such as CENTRAL and other trial registries, need to receive continued encouragement and support in order to eliminate the need for multiple-source search endeavors.

Over and above the recalls, the median precisions are quite low and indicate a need for improved indexing in databases. Efforts to improve and standardize the indexing of various databases need to be supported. Guidelines for journals and authors regarding the reporting of key methodological or subject terms when publishing studies would facilitate these efforts [22]. In addition to very poor precisions, the authors of our included studies reported precisions or the data necessary to calculate precision only 40% (47/117 comparisons) of the time.

Most of the research has involved MEDLINE and EMBASE, two of the major databases that the systematic review community recommends reviewers search. However, searching multiple databases can be difficult, time consuming and costly. For example, although MEDLINE is available freely on the Internet through PubMed, EMBASE is very costly and many institutions do not subscribe to it. This is of particular concern as studies have demonstrated that there is 17 to 75% overlap between MEDLINE and EMBASE [3,23,24] indicating that EMBASE may yield a substantial number of unique articles. To date, the gold standard for conducting systematic reviews still remains searching multiple bibliographic databases and hand searching. In addition, using only MEDLINE for systematic reviews still results in important trials being missed thereby compromising the external validity of the review.

Optimally, it would be most efficient to search few resources, retrieve a maximum yield of relevant trials, and retrieve a minimum yield of irrelevant trials. The Cochrane Collaboration is trying to achieve this with the Controlled Trials Register (CENTRAL) available through the Cochrane Library. The register now includes over 420,000 RCTs and CCTs. While there are numerous studies that discuss the vast amount of trials that have been added to CENTRAL through handsearching efforts, there are very few studies evaluating whether CENTRAL can be searched exclusively for RCT/CCTs. If one resource (e.g., CENTRAL) can be searched to identify RCT/CCTs, this would substantially reduce the time and costs associated with searching.

There was extensive heterogeneity among topics investigated in the studies included in this review. For the comparisons which had many studies, the values for both recall and precisions covered most of the possible range (e.g., 0–100). Thus, the topic searched may be the strongest determinant of the results. Topics are indexed differentially within and across various sources. Due to the between-study heterogeneity, very little can be concluded about the indirect subgroup results.

Over the 20 years that this review covers, it was noted that the older studies were conducted prior to indexing improvements in resources, especially MEDLINE. While there have been numerous changes in search technology in the past two decades, upon conducting post-hoc sub-group analyses, no difference was found. In addition, a sub-group analysis was done of recent studies and no difference was found when compared to the results of older studies. As mentioned above, this may be due to topic heterogeneity, not changing search technology. Thus, including the older studies did not confound our results and did not lead to the conclusion that there is one sufficient resource which identifies RCT/CCTs. Unfortunately, the more recent studies are not showing results which significantly differ from the ones obtained 20 years ago.

We found that, generally, both *Complex *and *Cochrane *search strategies performed better in recall than did *Simple *search strategies without loss in precision. However, the indirect subgroup results for recall showed support for this finding for the *Cochrane *search strategies, but not for the *Complex *search strategies. The *Cochrane *search strategy precisions were poorer in the indirect subgroup results, however data were sparse. Little direct evidence was available comparing searchers with different expertise. No direct evidence was available comparing searches for different design types. Without supporting direct subgroup evidence, conclusions from the indirect subgroup evidence would be too speculative. Other reasons that may explain the heterogeneity include: 1) the time period covered by the search (indexing as well as other search technologies have progressed over time which would affect the accuracy of searches); and 2) the methodological quality of the study. For example the rigor with which handsearching was done, searches were screened, or inclusion criteria applied (e.g., having 2 people independently perform each step) would affect the comparability of results across studies.

The quality of the included studies varied and, in most cases, the poor quality result was due to the lack of rigor in the reporting of the selection methodology. One-third reported that standard inclusion and exclusion criteria were developed and applied to each database/method at the relevance stage. Almost all studies did not indicate that at least 2 people independently screened the searches for potentially relevant studies. In addition, two-thirds of the included studies did not indicate that at least 2 people independently applied eligibility criteria to identify relevant studies. Quality of these studies can be improved by adopting more stringent methodology and reporting its use.

Post-hoc, we looked at how the calculated results of recall and precision may have improved over the last two decades considering the changes in search technology (in particular, indexing). Within our three MEDLINE comparisons, we found no pattern of association between year of publication and results. The correlations ranged from -0.91 to 1. As mentioned above, this quantitative analysis may be too dilute due to topic heterogeneity. We suggest a within topic analysis to robustly test for improvements in search technology.

This paper provides the most current and comprehensive review of the existing evidence comparing any electronic database against any other source or combination of sources. This and previous reviews demonstrate that there is a dearth of evidence regarding the use of different databases to retrieve RCTs, with the notable exception of handsearching and MEDLINE. Therefore, searching multiple resources to retrieve RCTs cannot be ruled out based upon this evidence. There needs to be more research done on major databases such as: EMBASE, CENTRAL, PsycINFO and trial registries in order to gather more information about the value of these databases in identifying RCT/CCTs for various topical areas. This review is more comprehensive than previous work in this area [8] and reflects the different ways that searches can be conducted (i.e., using a variety of databases and types of searches). Moreover, while a previous review focused upon between-study subgroup comparisons [8] (e.g., *Complex *versus *Simple *search strategies), we also systematically examined the within-study subgroup comparisons which provides more valid information [12]. However, similar to negative clinical trials, it is important recognize the limitations of current resources and the implications for decision-making.

## Limitations

There are several limitations to this study. Foremost, there is a lack of a validated quality score for this type of study (i.e., comparative). Reference standards are difficult to compare as they are generally different and may not be reported in enough detail to be reproducible. As well, the topic chosen to search can determine the success of the strategy. In addition, there are limitations to using precision and recall which are addressed by Kagolovsky and Moehr [25].

## Conclusion

### Implications for practice

Since recall is low with single resources, multiple-source comprehensive searches continue to be necessary. The *Cochrane *search strategy or *Complex *search strategy in consultation with a librarian are recommended.

### Implications for research

Efforts to enhance and build CENTRAL, a large trial registry, need to be continued. A number of the resources used to find trials for CENTRAL (e.g., journals, grey literature) are not indexed in MEDLINE, therefore CENTRAL has a significant amount of unique information not found in any other source. CENTRAL is also free for researchers in developing countries and available in CD-ROM and on the internet. Based upon the results of why studies were missed, indexing efforts also need to improve. Guidelines should be provided for authors to include MeSH terms and keywords in their abstracts which can then be used by indexers. Other resources that need to be studied include: Trial registries, LILACS, PsycINFO, Science Citation Index, BIOSIS, CABNAR and CINAHL. In addition, those researchers studying searches need to report precision results.

## List of abbreviations used

CCT – controlled clinical trial

RCT – randomized controlled trial

## Competing interests

The author(s) declare that they have no competing interests.

## Authors' contributions

ETC conceived of the study, designed and coordinated it and drafted the manuscript. NW performed the statistical analysis and drafted and read the final manuscript. LH and KC participated in the study and helped to draft the manuscript. TK participated in the study design and read the final manuscript. All authors participated in the design of the study and read and approved the final manuscript.

## Appendix 1: MEDLINE Search Strategy

1 medline.mp.

2 internet.mp.

3 embase.mp.

4 (psyclit or psycinfo or psychlit or psychinfo).mp.

5 "web of science".mp.

6 cinahl.mp.

7 sigle.mp.

8 "system for information on grey literature in europe".mp.

9 lilacs.mp.

10 excerpta medica.mp.

11 "science citation index".mp.

12 "science citation abstracts".mp.

13 scisearch.mp.

14 toxline.mp.

15 aidsline.mp.

16 cancerline.mp.

17 pubmed.mp.

18 grateful med.mp.

19 cabnar.mp.

20 "health star".mp.

21 healthstar.mp.

22 "current contents".mp.

23 "cochrane library".mp.

24 ("cochrane controlled trials register" or central or cctr).mp.

25 "database of abstracts of reviews of effectiveness".mp.

26 eric.tw.

27 "world wide web".mp.

28 dissertation$.mp.

29 thesis.mp.

30 "institute of scientific information".mp.

31 isi.mp.

32 "inside information plus".mp.

33 firstsearch.mp.

34 "international pharmaceutical abstracts".mp.

35 "biological abstracts".mp.

36 (dare and cochrane).mp.

37 pascal.tw.

38 or/1–36

39 search$.mp.

40 (handsearch$ or "hand search$").mp.

41 compar$.mp.

42 "manual search$".mp.

43 or/39–42

44 (controlled adj2 trial$).mp.

45 clinical trial$.mp.

46 (randomized controlled trial$ or randomised controlled trial$).mp.

47 (rct or cct).mp.

48 or/44–47

49 and/38,43,48

## Pre-publication history

The pre-publication history for this paper can be accessed here:



## Supplementary Material

Additional File 1 Characteristics of included studies, information about the studies used in this systematic review Click here for file
