# Supplementary material for: Which resources should be used to identify RCT/CCTs for systematic reviews: a systematic review
Source: BMC Med Res Methodol. 2005 Aug 10;5:24. doi: 10.1186/1471-2288-5-24 (PMC1232852; doi:10.1186/1471-2288-5-24)
Supplement: Additional File 1 — Characteristics of included studies, information about the studies used in this systematic review [file 1471-2288-5-24-S1.doc]

Characteristics of Included Studies

| **Study** | **Topic(s)** | **Objective(s)** | **Comparisons** | **Reference Standard** | **Search**  **Years & Type(s)** | **Included Study Design(s)** |
| --- | --- | --- | --- | --- | --- | --- |
| Dickersin[26]  1985  (USA) | Neonatal hyperbilirubinemia; intraventricular hemorrhage in newborns | To compare searches in MEDLINE to the gold standard Register of Controlled Trials in perinatal medicine. | MEDLINE search 1 vs. Trial registry vs. MEDLINE search 2 | Trial registry | 1966-85  *Simple & Complex search* | RCT, CCT |
| Poynard[27]  1985  (USA, France) | Hepatology (liver diseases) | To compare MEDLARS and hand searches for RCTs. | Handsearch vs. MEDLARS | Handsearch | 1966-82  *Complex search* | RCT, CCT |
| Bernstein[28]  1988  (USA) | Hepatology (liver diseases) | To re-evaluate Poynard & Conn’s study that compared MEDLARS and hand searches for RCTs. | Handsearch vs. MEDLARS | Handsearch | 1966-82  *Complex search* | RCT, CCT |
| Brand[29]  1999  (USA) | Hypertension | To calculate the sensitivity of the publication type *Randomized Controlled Trial* and of the Cochrane search strategy for identifying reports of RCTs in 4 major journals. | Handsearch vs. MEDLINE | Handsearch | 1979-96  *Simple & Cochrane search* | RCT |
| Kirpalani[21]  1989  (Canada) | Newborn care | Determine the sensitivity, specificity and positive predictive value of MEDLINE searches for RCTs of newborn infant care. | Handsearch vs. MEDLINE search 2 (Amateur search) vs. MEDLINE search 1 (Librarian search) | Handsearch | 1985  *Complex search* | RCT, CCT |
| Hofmans[30]  1990  (Netherlands) | Acupuncture | To investigate publication bias by analyzing references from a meta-analysis. | Meta-analysis vs. MEDLINE vs. Index Medicus vs. Gold standard | Meta-analysis references | 1966-1989  *Simple search* | RCT |
| Gøtzsche[31]  1991  (Denmark) | Rheumatoid arthritis | To test different MEDLINE search strategies for RCTs. | MEDLINE search 1 (comparative study) vs. MEDLINE search 2 (double-blind method) | MEDLINE, pharmaceutical companies, references | 1966-85  *None & simple search* | RCT, CCT, double-blind trials |
| Kleijnen[32]  1992  (Netherlands) | Homeopathy; ascorbic acid for common cold; gingko biloba for claudication and cerebral insufficiency | To compare electronic and handsearching. Are there differences between identified articles and other publications from non-indexed sources. | Handsearch vs. MEDLINE vs. EMBASE | MEDLINE, EMBASE, Current Contents, references, conference proceedings, journals, personal communication, pharmaceutical companies, visiting special libraries, word of mouth | 1940-91  *Complex search* | CCT |
| Jadad^a^[33]  1993  (UK) | Anesthetic; pain | To produce estimates of the time involved in identifying RCTs published in specialist journals, MEDLINE & Handsearch. To determine the sensitivity and precision of MEDLINE searches for RCTs. To determine the causes of missed articles, efficient combinations of MEDLINE and selective handsearching to achieve high sensitivity at minimal cost. | Handsearch vs. MEDLINE vs. Supplement issues | Handsearch + MEDLINE | 1966-92  *Complex search* | RCT, CCT |
| Jadad^b^[34]  1993  (UK) | Primary care | To refine [13] search strategy to identify more reports on pain relief. | Handsearch vs. MEDLINE | Handsearch + MEDLINE | 1966-91  *Complex search* | RCT |
| Silagy[13]  1993  (UK) | Primary care | To determine the number, nature, site of publication and feasibility of identifying RCTs. | Handsearch vs. MEDLINE | Handsearch + MEDLINE | 1968-91  *Complex search* | RCT |
| Adams[35]  1994  (UK) | Mental health care | To identify sensitivity and precision of MEDLINE and handsearch for RCTs. | Handsearch vs. MEDLINE search 1 (standard search) vs. MEDLINE search 2 (key search) vs. MEDLINE search 3 (Cochrane skilled search) | Key, standard, skilled and hand searches | 1971, 1976, 1981, 1986, 1991  *Simple & Complex search* | RCT, CCT |
| Aker[36]  1994  (Canada) | Manual therapy to the cervical spine | To determine whether searching computerized databases in addition to MEDLINE would yield further relevant literature. To compare the cost and efficiency of four different databases. | Chirolars vs. CINAHL vs. EMBASE vs. MEDLINE | Chirolars + CINAHL + EMBASE + MEDLINE | 1966 onwards  *Complex search* | RCT |
| Dickersin[37]  1994  (USA) | Ophthalmology | To determine the sensitivity and precision of MEDLINE searches for randomized clinical trials. To develop a register of trials. | Handsearch vs. MEDLINE search 1 (broad search) vs.  MEDLINE search 2 (sensitive search) | Handsearch + MEDLINE | 1988-89  *Cochrane search* | RCT, CCT |
| Solomon[38]  1994  (Canada) | General surgery | To determine RCTs in 1990; the area, country, centers involved, are surgeons principal authors, funding, journals published in, therapy being tested & assess study quality | Handsearch vs. MEDLINE search 1 (publication type + MeSH) vs. MEDLINE search 2 (random & double-blind) | Handsearch | 1990  *Simple search* | RCT |
| Tsutani[14]  1994  (Japan) | Japanese randomized controlled trials | To conduct a comprehensive analysis of the content and accessibility of three Japanese databases and MEDLINE. | JAPICDOC vs. JMEDICINE vs. JICST-E vs. MEDLINE | JAPICDOC + JMEDICINE + JICST-E | Years not stated  *Index Search?* | RCT |
| Johnson[39]  1995  (USA) | No topic (study designs only) | To compare the numbers of items retrieved from MEDLINE and full-text NEJM and examine the unique access points available in each. | MEDLINE vs. NEJM | NEJM database | 1990-94  *Simple search* | RCT, other trials |
| Clarke^a^[40]  1996  (UK) | American Society of Clinical Oncology | To compare a handsearch of the 1,471 abstracts from the 1992 ASCO meeting with an indexed bibliographic database to identify RCTs. | Handsearch vs. CancerLIT search 1 vs. CancerLIT search 2 vs. Handsearch + CancerLIT | Handsearch | Year not stated  *Simple search* | RCT |
| Clarke^b^[41]  1996  (UK) | Lancet | To identify the numbers of reports of RCTs correctly tagged by the National Library of Medicine and the number of additional reports identified by electronic search and handsearch. | MEDLINE search 1 vs. MEDLINE search 2 vs. MEDLINE search 3 | Handsearch + MEDLINE | 1994  *Cochrane search* | RCT |
| Jadad[42]  1996  (UK and Canada) | Pain; analgesic interventions | To describe development, methods used to identify eligible RCTs & management of information in pain research from 1950-90; estimate time cost of each step in the process | Handsearch vs. MEDLINE | Handsearch + MEDLINE | 1950-90  *Complex search* | RCT, CCT |
| Hay[43]  1996  (UK) | Eating disorders | To identify RCTs by handsearch and compare to MEDLINE & EMBASE. | Handsearch vs. EMBASE vs. PsycLIT | Handsearch | 1981-93  *Complex search* | RCT, CCT |
| Jefferson[44]  1996  (UK) | Vaccine trials | To identify trials in a journal by handsearch. To set up a database of trials. | Handsearch vs. MEDLINE | Handsearch | 1983-94  *Cochrane search* | RCT, CCT |
| Marson[45]  1996  (UK) | Epilepsy | To compare MEDLINE search strategies to a handsearch. To look at why articles missed in MEDLINE search. | Handsearch vs. MEDLINE | Handsearch | 1966-93  *Simple & Complex search* | RCT, CCT |
| McDonald[46]  1996  (UK) | BMJ; Lancet | To handsearch the BMJ and Lancet from 1948-94 to identify RCTs and CCTs. | Handsearch vs. MEDLINE | Handsearch | 1948-94  *Simple & Cochrane search* | RCT, CCT |
| Neal[47]  1996  (New Zealand) | New Zealand Medical Journal | To identify RCTs; to document characteristics of trials and count the number in MEDLINE. | Handsearch vs. MEDLINE | Handsearch | 1943-95  *Cochrane search* | RCT, CCT |
| Spoor[48]  1996  (UK) | Diabetic medicine | To find RCTs in Diabetic Medicine from 1984-94 by handsearch & MEDLINE | Handsearch vs. MEDLINE | Handsearch + MEDLINE | 1984-94  *Simple search* | RCT, CCT |
| Veldhuyzen van Zanten[17]  1996  (Canada/ Australia/ Sweden/USA/  Belgium/ Netherlands) | Functional dyspepsia | To evaluate drug therapy of functional dyspepsia and provide guidelines for trials | Handsearch vs. MEDLINE search 1 (dyspepsia & random allocation) vs. MEDLINE search 2 (dyspepsia & therapeutics) | Handsearch + MEDLINE | Handsearch 1980-?  1966-93  p. 661 | RCT |
| Bara[49]  1997  (UK) | Airways | To test the sensitivity of handsearching full-text searching for the identification of RCTs. | Handsearch vs. MEDLINE vs. Gold standard | Handsearch + MEDLINE | 1970-95 (select years)  *Complex search* | RCT |
| Bender[50]  1997  (Canada) | Obstetrical anaesthesia | To determine RCTs published and their quality. Compare a broad MEDLINE search strategy to a handsearch. | Handsearch vs. MEDLINE search 1 (MeSH) vs.  MEDLINE search 2 (MeSH + text terms) | Handsearch | 1985-94  *Simple & Complex search* | RCT, CCT |
| Cullum[51]  1997  (UK) | Nursing | To find nursing RCTs in nursing by developing an electronic search strategy and hand searching. To make the RCTs available through the MEDLINE enhancement programme and by publication in the Cochrane controlled trials register. To identify existing systematic reviews. | Handsearch vs. MEDLINE | Handsearch + MEDLINE | 1987-94  *None & Cochrane search* | RCT, CCT, SR |
| Duggan[52]  1997  (UK) | Journal of Intellectual Disability Research (learning disability) | To identify and describe all RCTs. To compare the ease of idenfication of the RCTs in MEDLINE and PsycLit. | Handsearch vs. PsycLIT vs. MEDLINE search 1 (author performed) vs. MEDLINE search 2 (based upon Cochrane search) | Handsearch | 1957-94  *None & Cochrane search* | RCT, CCT |
| McDonald[53]  1997  (UK) | General health care | To compare the reports of controlled trials identified by handsearching with those identified by the Cochrane highly sensitive search strategy. | Handsearch vs. MEDLINE | Handsearch | 1996  *Simple & Cochrane search* | RCT, CCT |
| Pasternack[54]  1997  (Finland) | Medical journals in Finland | To determine the number of RCT, CCT and meta-analyses published in main medical journals in Finland. To assess the sensitivity and precision of handsearch and electronic (MEDLINE) search. | Handsearch vs. MEDLINE | Handsearch + MEDLINE | 1954-96  *Cochrane search* | RCT, CCT, MA |
| Reynolds[55]  1997  (USA) | General medical journals | To compare the sensitivity and yield of electronic searching and handsearching for randomized controlled trials and non-randomized, controlled clinical trials for 11 US general medical journals for 1970, 1981, and 1992. | Handsearch vs. MEDLINE vs. Gold standard | Handsearch | 1970, 1981, 1992  *Cochrane search* | RCT, CCT |
| Smith[56]  1997  (Canada) | Community health interventions | To determine if RCT methodology used appropriately; to compare hand with MEDLINE search for RCTs. | Handsearch vs. MEDLINE | Handsearch | 1992  *Simple search* | RCT, NRIS |
| Ahmed[57]  1998  (US) | Archives of General Psychiatry | To handsearch 1959-95 of the Archives of General Psychiatry and note information about each RCT identified. | Handsearch vs. MEDLINE | Handsearch | 1959-95  *None* | RCT |
| Gluud[58]  1998  (Denmark) | Hepatology | To handsearch the Journal of Hepatology, identify all fully reported RCTs and make a qualitative assessment of their reporting. | Handsearch vs. MEDLINE | Handsearch + MEDLINE | 1985-97  *Cochrane search* | RCT, CCT |
| Nwosu[59]  1998  (UK) | Obstetrics & gynecology | To develop and test a MEDLINE search strategy to identify RCTs | Handsearch vs. MEDLINE | Handsearch + MEDLINE | 1975, 1980, 1985, 1990, 1995  *Simple search* | RCT, CCT |
| Thomas[15]  1998  (UK) | Stroke | To determine the number of stroke trials identified in 5 different databases, the degree of duplication and the number of unique trials found in the authors’ specialized register. | MEDLINE vs. EMBASE vs. Biosis vs. DERWENT Drug File vs. SciSEARCH | Not stated | Years not stated  *Complex search* | Trials |
| Brazier[60]  1999  (UK) | Pre-hospital care | To identify RCTs; to identify the themes considered in these RCTs; to compare the sensitivity and precision of the MEDLINE and EMBASE databases. | MEDLINE vs. EMBASE | MEDLINE + EMBASE | 1987-97?  *Cochrane search* | RCT, CCT |
| Croft[61]  1999  (UK) | Journal of the Royal Army Medical Corps | To handsearch the Journal of the Royal Army Medical Corps for RCT/CCTs from 1948-1998. | Handsearch vs. MEDLINE | Handsearch | 1990-98  *Cochrane search* | RCT, CCT |
| Langham[62] 1999  (UK) | Emergency medicine | To identify RCTs. To compare motives for participation in handsearching by emergency medicine professionals; to compare handsearching with MEDLINE searching. | Handsearch vs. MEDLINE vs. Handsearch only | Handsearch | 1948-95  *Cochrane search* | RCT, CCT |
| MartÍ[63]  1999  (Spain) | General and internal medicine | To locate all controlled clinical trials published in Spanish journals of general medicine, between 1971 and 1995, to characterize them, and to incorporate them into the worldwide database of clinical trials maintained by the Cochrane Collaboration. | Handsearch vs. MEDLINE | Handsearch | 1971-95  *Cochrane search* | CCT |
| Schlomer[64]  1999  (Germany) | Nursing | To ascertain whether there are German RCTs and systematic reviews in nursing. Quantitative comparison of German and international nursing research. | Handsearch vs. MEDLINE vs. CINAHL | Handsearch | 1988-97  *Complex search* | RCT, CCT, SR |
| Watson^a^[65]  1999  (UK) | Group psychotherapy | To investigate the adequacy of MEDLINE and PsycINFO in retrieving articles describing outcome studies of group-based psychological treatments. | Handsearch vs. MEDLINE vs. PsycINFO | Handsearch + MEDLINE + PsycINFO | 1993-94  *Complex search* | RCT |
| Watson^b^[66]  1999  (UK) | Cognitive therapy for depression | To compare EMBASE, MEDLINE and PsycINFO in retrieving RCTs. To compare different search strategies. | EMBASE vs. MEDLINE vs. PsycINFO | EMBASE, MEDLINE + PsycINFO | 1992-96  *Complex search* | RCT |
| Bassler[67]  2000  (Germany) | Pediatrics | How many CCTs are published in 3 German pediatrics journals in the past 30 years? What are the characteristics of these trials concerning their methodological quality and their representation in MEDLINE? | Handsearch vs. MEDLINE | Handsearch | 1970-98  *Simple search* | RCT, CCT |
| Adetugbo[68]  2000  (UK) | Dermatology | To assess methodological quality of the design and reporting of RCTs. | Handsearch vs. MEDLINE | Handsearch | 1976-97  *None* | RCT, CCT |
| Bereczki[69]  2000  (Hungary) | Neurology; neurosurgery ; psychiatry; clinical neurosciences | To determine if a journal not indexed in MEDLINE has RCTs or CCTs not also reported in other MEDLINE publications. | Handsearch vs. MEDLINE search (family name & first initial) vs. MEDLINE search (family name & disease intervention) | Handsearch | 1950-98  *Simple search* | RCT, CCT |
| Dumbrigue[70] 2000  (USA) | Prosthodontics | Evaluate sensitivity, specificity and precision of MEDLINE search strategies for RCTs. | Handsearch vs. MEDLINE | Handsearch | 1988-97  *Complex search* | RCT |
| Fergusson[20] 2000  (Canada / France) | Allogeneic blood transfusion in elective surgery | Explore type of literature search strategy, inclusion or exclusion of abstracts, letters or conference proceedings; the inclusion or exclusion of non-english language publications; the effect of trial quality upon the results of meta-analyses and the inclusion or exclusion of non-placebo-controlled studies | MEDLINE vs. EMBASE | MEDLINE, EMBASE, authors + references | 1966-96  *Simple, Complex & Cochrane search* | RCT |
| Galandi[71]  2000  (Germany) | General health care | To determine the value of MEDLINE and EMBASE for the identification of clinical trials published in German general health care journals. | Handsearch vs. MEDLINE search 1 vs MEDLINE search 2 vs. EMBASE search 1 vs. EMBASE search 2 | Handsearch | 1948-98  *None & Simple search* | RCT |
| Kennedy[19]  2000  (USA) | AIDS | To compare RCTs identified by MEDLINE with those identified through a handsearch of articles in the journal AIDS. | Handsearch vs. MEDLINE search 1 vs. MEDLINE search 2 | Handsearch | 1987-99  *Index search* | RCT |
| Olesen[72]  2000  (Denmark) | Scandanavian Journal of Rheumatology | To identify all reports of RCTs in a journal and compare with a MEDLINE search. | Handsearch vs. MEDLINE | Handsearch | 1955-97  *None* | RCT, CCT |
| Suarez[3]  2000  (USA, Canada) | Rheumatoid arthritis, low back pain and osteoporosis | To compare the retrieval performance of MEDLINE and EMBASE for CCTs. | Handsearch vs. MEDLINE vs. EMBASE | MEDLINE + EMBASE | 1988, 1994  *Cochrane search* | CCT |
| Avenell[73]  2001  (UK) | Nutrition | To evaluate the search plan for identifying RCTs in nutrition as part of a systematic review of nutritional supplementation trials in patients after hip fracture. | Handsearch + MEDLINE + EMBASE + CINAHL + CABNAR + BIOSIS + HealthStar | Handsearch + MEDLINE + EMBASE + Cinahl + CABNAR + BIOSIS + HealthStar | 1966-2000  *Complex search* | RCT, CCT |
| Eysenbach[74] 2001  (Germany) | Preterm premature rupture of membranes; severe mental disorders; asthma; obstructive sleep apnoea; retinopathy of prematurity; respiratory distress syndrome | Are searches on the world wide web useful to identify additional unpublished and ongoing clinical trials. To develop and evaluate a search strategy for finding trials on the internet | Internet vs. Systematic reviews | Included studies of 3 systematic reviews | 1998-2000 (internet search on AltaVista)  *Simple search* | RCT, CCT |
| Helmer[18]  2001  (Canada) | Acupuncture for addiction, lipid-lowering drug therapy for coronary heart disease | To test effectiveness of systematic search strategies to identify RCTs. | Handsearch vs. Major databases vs. Extended methods | Major databases + extended search | No years listed  *None* | RCT |
| Furukawa[75]  2002  (Japan, UK) | Depression; neuroleptics | To examine the Cochrane Depression, Anxiety and Neurosis Group and Cochrane Schizophrenia Group’s trial registries against a register of Japanese trials. | Handsearch vs. Trial registry 1 vs. Trial registry 2 vs. Trial registry 3 | Japanese registry + CCDAN | 1981-2000  *Simple search* | RCT |
| Hopewell[76]  2002  (UK) | Specialized health care journals | To compare MEDLINE & hand searches to identify RCTs. To submit handsearch trials to CENTRAL and MEDLINE for retagging. | Handsearch vs. MEDLINE | Handsearch | 1970-1999  *Simple search* | RCT, CCT |
| Isaakidis[77]  2002  (Greece) | Disease burden | To evaluate whether the amount of randomised clinical research on various medical conditions is related to the burden of disease and health needs of the local populations in sub-Saharan Africa. | MEDLINE vs. CENTRAL vs. African Published Trials Register of the South African Cochrane Center | MEDLINE + Central + African Trial Registry | 1966-2000  *Simple & Cochrane search* | RCT |
| Langham[78]  2002  (UK) | Critical care | To compare handsearching with MEDLINE searching (employing a comprehensive search strategy) in 15 critical care journals and to assess the quality of reporting of RCTs. | Handsearch vs. MEDLINE | Handsearch + MEDLINE | 1948-95  *Cochrane search* | RCT, CCT, possible RCT |
| Royle[79]  2003  (UK) | New reviews in CDSR | To determine the sources currently used in Cochrane reviews to search for trials.  Measure the proportion of trials indexed in 4 databases.  Analyze RCTs not indexed in CCTR, MEDLINE or EMBASE.  Compare the quality of trials found in CCTR, MEDLINE or EMBASE with those from other sources. | CENTRAL vs. MEDLINE vs. EMBASE vs. Science Citation Index/Social Sciences Citation Index | whatever the systematic reviews searched | Years not listed  *Simple search* | RCT, CCT |
| Sampson[1]  2003  (Canada) | Meta-analysis | Does EMBASE provide material not in MEDLINE for meta-analysis? | EMBASE vs. MEDLINE | MEDLINE + EMBASE + grey literature | 1966-2000  *Simple search* | RCT |
